# Interdisciplinary interventions in the perioperative rehabilitation of total laryngectomy: an integrative review

**DOI:** 10.6061/clinics/2018/e484s

**Published:** 2018-08-28

**Authors:** Vitor Modesto Rosa, Joselia Maria Lira Fores, Erika Priscila Ferreira da Silva, Elizeteh Oliveira Guterres, Aline Marcelino, Paula Cristina Nogueira, Wania Regina Mollo Baia, Marco Aurélio Vamondes Kulcsar

**Affiliations:** I Servico de Nutricao e Dietetica, Instituto do Cancer do Estado de Sao Paulo (ICESP), Hospital das Clinicas HCFMUSP, Faculdade de Medicina, Universidade de Sao Paulo, Sao Paulo, SP, BR; II Enfermagem, Instituto do Cancer do Estado de Sao Paulo (ICESP), Hospital das Clinicas HCFMUSP, Faculdade de Medicina, Universidade de Sao Paulo, Sao Paulo, SP, BR; III Fisioterapia, Instituto do Cancer do Estado de Sao Paulo (ICESP), Hospital das Clinicas HCFMUSP, Faculdade de Medicina, Universidade de Sao Paulo, Sao Paulo, SP, BR; IV Endoscopia, Instituto do Cancer do Estado de Sao Paulo (ICESP), Hospital das Clinicas HCFMUSP, Faculdade de Medicina, Universidade de Sao Paulo, Sao Paulo, SP, BR; V Escola de Enfermagem EEUSP, Universidade de Sao Paulo, São Paulo, SP, BR; VI Diretoria de Assistência, Hospital Sirio-Libanes, São Paulo, SP, BR; VII Cirurgia de Cabeca e Pescoco, Instituto do Cancer do Estado de Sao Paulo (ICESP), Hospital das Clinicas HCFMUSP, Faculdade de Medicina, Universidade de Sao Paulo, Sao Paulo, SP, BR

**Keywords:** Patient Care Team, Laryngectomy, Medical Oncology

## Abstract

The aim of this study was to use the scientific literature to identify interdisciplinary interventions for rehabilitation during the perioperative period for cancer patients who underwent total laryngectomy. We systematically researched controlled descriptors: laryngectomy, patient care team/education, patient care team/manpower, patient care team/methods, patient care team/utilization and rehabilitation. We performed a qualitative narrative synthesis and identified 549 articles. Of these, 113 were duplicates, 398 were excluded during the analysis of the title and abstract, 1 was excluded for unfeasibility of access, and 4 were excluded after reading the article in full, resulting in 33 included articles. The articles addressed different types of interdisciplinary actions, such as vocal, olfactory, pulmonary and alimentary rehabilitation; comparisons of prosthetic devices; and descriptions of practices for total laryngectomized patient rehabilitation. Although the interventions found in the literature were effective in the rehabilitation of the total laryngectomized patient, their interdisciplinarity was not evidenced but was highlighted in these studies as a factor for improvement in terms of practical assistance and quality of life.

## INTRODUCTION

The treatment of laryngeal cancer is differentiated according to the disease stage. For stages I and II, maintenance of functionality, with the objective of guaranteeing voice quality and swallowing capacity, is prioritized. Thus, the treatments indicated are radiotherapy and surgery, which result in a 5-year patient survival rate of more than 90%. However, radiotherapy is more often indicated because it presents better results in the maintenance of functionality 1.

For stages III and IV, the chosen treatment is related to the modality that allows a longer disease-free time and/or the absence of orotracheal interventions (tracheostomy or tube feeding) for two years or more. Therefore, the choice of treatment depends more on the patient's ability to participate actively in rehabilitation than on the chosen treatment modality 2.

The surgical treatment takes into account the different portions and extent of involvement of the laryngeal tumor and is basically divided into partial and total laryngectomy, which differ mainly in terms of the anatomical alteration performed because when the structures that allow the recovery of breathing and phonation are maintained, a better quality of life for the patient and return to social activities is enabled 3.

In the total laryngectomy procedure, the three laryngeal (supraglottic, glottic and infraglottic) portions of the cartilaginous skeleton, the pre-epiglottic space and the laryngeal musculature are removed. As a consequence, a definitive tracheostoma is necessary, since there is no further communication between the lungs and upper airways, making phonation impossible 3.

At the same time, late side effects of treatment may occur, such as dental and oral health complications (xerostomia, osteoradionecrosis, trismus, dysphagia, tube feeding, neuromuscular toxicity and speech difficulties), recurrent laryngeal nerve palsy, chronic pain, ototoxicity, peripheral neuropathy, autonomic dysfunction, musculoskeletal complications, damage to the carotid arteries, nephrotoxicity and psychosocial issues 4.

Complications can be minimized during rehabilitation, which can be defined as a set of therapeutic measures aimed at the individual reaching the maximum of his physical, psychological and social capacity, using preventive, therapeutic, adaptive and palliative measures 3. In oncology, context distinguishes these patients from others, since these patients are more susceptible to intercurrences and worsening of their general state; in addition to an agenda of rehabilitation therapies, their care often comprises sessions of radiotherapy, chemotherapy and frequent medical consultations 3.

In this context, an interdisciplinary approach is necessary because the treatment depends on the extent and location of the tumor; the patient's age, performance status, comorbidities, and psychosocial support; and the availability of a rehabilitation service. The main objective of the rehabilitation service is to perform an evaluation that is integral to the needs of the patient, in addition to providing the rehabilitation care line and addressing specific issues of each of the specialties, with the richness of effective interdisciplinarity 2,3.

The interdisciplinary approach in vocal rehabilitation after laryngectomy should cover the perioperative period to produce the best speech results. The vocal restoration of the post-laryngectomy patient is a complex task and usually occurs throughout the patient's lifetime. The best results are found with pre-operative interdisciplinary planning, which includes robust pre-operative educational efforts, a meticulous and safe surgical technique that supports hospital care, postoperative follow-up that includes permanent education and speech therapy 5.

Thus, adherence to rehabilitation is a fundamental factor in the treatment of laryngeal cancer patients, where each professional performs specific rehabilitation interventions aiming at a better quality of life for the patient and family 2,6.

In view of the above, this study aimed to evaluate the scientific knowledge produced on the subject, seeking to identify the interdisciplinary interventions for the perioperative rehabilitation of cancer patients who underwent total laryngectomy.

Therefore, the objective of this study was to identify, through the scientific literature, interdisciplinary rehabilitation interventions during the perioperative period for cancer patients who underwent total laryngectomy.

## MATERIALS AND METHODS

This is an integrative review, which uses the acronym PICO, where P indicates population, I indicates intervention, and CO indicates context 7 and where P = oncology patients who underwent total laryngectomy, I = interdisciplinary interventions, and CO = perioperative rehabilitation. Thus, the research question was as follows: What are the interdisciplinary interventions for perioperative rehabilitation for cancer patients who undergo total laryngectomy?

The inclusion criteria were studies in the Spanish, English and Portuguese languages, studies performed from 2010 to 2015, and studies with adult oncology patients and/or elderly patients undergoing perioperative rehabilitation after total laryngectomy who participated in rehabilitation interventions performed by a multiprofessional team. We excluded studies performed on animals, children and patients with partial or subtotal laryngectomy.

The selection of articles for the review occurred in 4 steps: identification, screening, eligibility and inclusion. The identification stage comprised the bibliographic search that occurred from November 2015 to January 2016, and the studies were identified with the following databases: Cumulative Index to Nursing and Allied Health Literature (CINAHL), Cochrane Central Register of Controlled Trials (CENTRAL) from The Cochrane Library, National Library of Medicine/NLM (MEDLINE)/PubMed and Biomedical Database (EMBASE). A manual search was also carried out, through a reference consultation of articles related to the topic and contact with researchers working in the area of rehabilitation. The search strategies were formulated according to the criteria and manuals of each database. The following descriptors were used: *Medical Subject Heading* (MeSH), Health Sciences Descriptors (DeCS) and terms related to the research problem (cancer rehabilitation) combined with Boolean operators (AND e OR) and truncation symbols. Table 1 presents the adopted search strategy, which was adapted according to each analyzed database.

For the screening stage, the studies were evaluated by title and summary independently by two reviewers, who applied the eligibility criteria and selected the relevant studies. For the subsequent eligibility stage, the selected studies were evaluated in full with the application of the inclusion and exclusion criteria, and in the last step, namely, the inclusion stage, the studies that were included and excluded from the review were defined.

To extract the data, the authors used an Excel spreadsheet, which included the numerical identification of the publication, title, authors, journal title and year of publication, objectives, method used, and results, namely, the interventions of the multiprofessional team in the rehabilitation of patients and conclusions/recommendations and level of evidence. The level of evidence (Table 2) of the studies was analyzed through the proposal of Melmyk and Fineout-Overholt 8.

## RESULTS

A total of 549 articles were found. Of these, 113 were duplicates, 398 were excluded in the title and abstract analysis, 1 was excluded for unfeasibility of access, and 4 were excluded after reading the article in full, bringing the total number of articles for analysis in the review to 33 (Table 3).

Regarding the methodological quality of the studies, most of them were descriptive or qualitative and were classified as having a level of evidence VI, which is considered weak (Table 4).

Regarding the objective of this review, i.e., interdisciplinary rehabilitation interventions in cancer patients during the perioperative period of total laryngectomy, actions were identified in relation to vocal rehabilitation (8 studies), olfactory methods (6 studies), pulmonary methods (5 study) (1 study), comparisons of prosthetic devices (3 studies) and descriptions of total laryngectomy rehabilitation practices (10 studies).

Most of the articles were written by physicians 24, followed by a physiotherapist 1, a nurse 1, a speech therapist 1, an occupational therapist 1 and a psychologist 1; in 4 studies, identifications of the profession of the author were not possible.

### Vocal rehabilitation

The vocal rehabilitation interventions found in the review of the literature were voice by tracheoesophageal prosthesis, esophageal voice and voice by electrolarynx. Additionally, we found interventions that contribute to the improvement of voice quality in previous models.

Voice restoration with the tracheoesophageal prosthesis is performed by surgical implantation of a device between the trachea and the esophagus, a mechanism that is basically a unidirectional valve, which allows the passage of the pulmonary airflow into the esophagus through the digital occlusion of the prosthesis. The new sound source is the pharyngoesophageal segment, which consists of the constricting pharynx and cricopharyngeal muscles and the upper portion of the esophagus, which vibrates and produce the sound 9-11.

The implantation of the tracheoesophageal prosthesis for vocal rehabilitation may occur soon after total laryngectomy or late because of the risk of serious postoperative complications, such as fistula formation or wound failure. However, regardless of the time of implantation, the results of voice rehabilitation are similar 9,12.

Digital occlusion of the prosthesis during speech is still regarded as a major drawback of the tracheoesophageal prosthesis. The development of a valve with automatic tracheostomy closure facilitates hands-free speech, which presents better results when fixed with a silicone adhesive and external cervical collar 13.

The esophageal voice is characterized by the momentary introduction of air into the esophagus and its ejection, causing the vibration of the pharyngoesophageal segment, a segment that becomes the new sound source, to produce the voice 10.

The pharyngoesophageal segment may present spasms resulting from surgery, being one of the causes of apophony and poor quality of the esophageal voice and tracheoesophageal prosthesis. The botulinum toxin type A technique, which involves injecting the toxin into the pharyngoesophageal segment, blocks the presynaptic release of acetylcholine at the neuromuscular junction, preventing hyperactive nerve impulses that trigger excessive muscle contractions, allowing better vibration of the segment and better voice quality 10,14.

The electrolaryngeal voice has the hypopharynx as the sound source. The electrolaryngeal voice produces the sound through the oscillator device that is placed on the wall of the neck and captures the vibration of the hypopharynx walls, producing the sound 9.

Regardless of the type of vocal rehabilitation, patients who were trained at admission or in the outpatient clinic presented better results than those who did not perform any training 15. In addition to training, the dedication of a patient and speech pathologist is necessary for adequate performance 16.

### Olfactory rehabilitation

Two olfactory rehabilitation interventions were found: a) the laryngeal shunt device and b) the polite yawning technique 17.

The laryngeal bypass device uses a flexible tube with a sealing lip around the stoma, with the other end held between the lips, temporarily connecting the mouth and nose to the lower airway. Thus, the natural pulmonary inspiration draws air through the nose before passing it through the mouth, tube and trachea. The patient should learn to relax the soft palate so that it does not obstruct the nasopharynx 17.

The polite yawning technique or nasal airflow-inducing maneuver utilizes the orofacial musculature to develop the means of generating nasal flow. It consists of a prolonged yawning movement with a simultaneous reduction in the mandible, floor of the mouth, tongue, base of the tongue and soft palate, keeping the lips tightly closed, producing nasal flow 17-19.

Olfactory rehabilitation with the polite yawning technique presents effective results both with the training by the professional and at home without support, but in the short term, the result is better when assisted by the professional 20.

### Pulmonary rehabilitation

Disconnection of the upper lower limb pathway prevents the heating, humidification and filtration of inhaled air, which can lead to lung problems. These can be compensated with the use of a heat exchanger and humidity 21-25.

### Food rehabilitation

In the case report of a patient with uncorrected pharyngocutaneous fistula in the various repair attempts with skin grafts, the bilateral major pectoralis muscle and deltopectoral myocutaneous flaps affected alimentary dependence via the gastric feeding tube. For alimentary rehabilitation, the salivary tube technique was used, which is the insertion of a bypass tube that connects the remaining funnel of the base of the tongue to the distal tube of the esophagus. This process transfers the saliva from the oral cavity to the esophagus, controlling salivary secretion and preventing the occurrence of aspiration, even with general diet consumption 26.

## DISCUSSION

In the field of oncology, there are many studies on the treatment of neoplasias, but few of them describe rehabilitation in an interdisciplinary way. The evidence-based practice is broad in terms of new techniques and the performance of each professional independently 27. Nevertheless, a series of studies published by *Current Opinion in Otolaryngology & Head & Neck Surgery* provides an enlightened and innovative method for rehabilitation of laryngectomized patients in several countries, and all of these studies emphasize the importance of the interdisciplinary approach.

In this series, interestingly, the need of each patient varies with culture, and with this variation, the quality of life assessment also has certain distortions. In the United States, the onset of preoperative rehabilitation is a distinctive feature that educates, informs, and empowers the patient through figures and illustrations and simple explanations of injury and treatment 5. This practice was not observed in any other study.

Studies related to the perioperative rehabilitation of total laryngectomies emphasize vocal, olfactory, pulmonary and alimentary restoration, as well as resources with technology to promote better adaptability to daily functional activities, social integration and autonomy for quality of life. To do this, these approaches rely on speech-language pathology, which depends on the conditions of each country, and can result in the addition of other professionals to the team 3,5,28-34.

In India, a study evaluated the impact of laryngectomy according to the country's socioeconomic conditions and observed the lack of specialized staff and centers for this type of rehabilitation 29. On the other hand, in the Netherlands, rehabilitation is carried out in multiprofessional centers involving medical professionals, nurses, speech therapists and psychologists for the purpose of vocal, olfactory, pulmonary and swallowing and palate rehabilitation 32.

The studies with the best levels of evidence (II and III) that were found in this review emphasize the importance of vocal rehabilitation, the maintenance of pulmonary health (due to the mechanical alterations consequent to surgery) and olfactory rehabilitation. For the reestablishment of oral communication, tracheoesophageal prosthesis with or without the use of a heat and moisture exchanger is recommended by a large number of reports 9,13,15,20,22-25,35-38.

A review that analyzed the practice of laryngectomized rehabilitation in Hong Kong highlights the conclusion that alaryngeal speech modalities do not satisfy the demand of the population that speak Cantonese because the approach does not allow changes in tones during speech, which are important for proper expression in this language 28.

The voice through the electrolarynx presents greater ease of rehabilitation but with lower patient satisfaction. In contrast, the esophageal voice is the most used in developing countries due to the low cost but has a lower success rate. Voice restoration with the tracheoesophageal prosthesis results in better satisfaction and self-confidence and minimizes the patient's social constraints, generating positive effects on quality of life, in addition to being more heavily used in developed countries 11,39,40.

A study comparing healthy people to people who underwent vocal rehabilitation with a prosthesis or esophageal speech identified a statistically significant difference in quality of life after their analysis of social function 11. This study proposes that the tracheoesophageal prosthesis can be considered a gold standard for speech rehabilitation 11,16,34,39. In addition, this study regards the esophageal voice as the second treatment option 11.

At the Cancer Institute of the State of São Paulo (ICESP), the continuity of vocal rehabilitation is stimulated with a choir group guided by a speech therapist for patients with an esophageal voice, a tracheoesophageal prosthesis or an electrolaryngeal voice. During the sessions, the presented difficulties are addressed individually but with the common goal of acquiring greater intelligibility, rhythm and greater respiratory control during esophageal speech 3.

The tracheoesophageal prosthesis and heat and moisture exchanger for voice restoration are presented as better resources in vocal and pulmonary rehabilitation, respectively; however, both are associated with a high cost that makes their incorporation into public health units difficult 39.

For olfactory rehabilitation, the polite yawning technique appears to be a good option because it is easily taught and learned and because it has a low cost, requiring only the professional specialized in the education of the patient. An important part of functional recovery that is most frequently mentioned in studies that assess quality of life is the olfactory capacity of the patient 19,32.

Failure to perceive bodily odor increases social insecurity, decreasing appetite, libido, sexual activity and the quality of the patient's mood, so olfactory rehabilitation with the polite yawning technique as an integral part of the post-laryngectomy rehabilitation program improves the quality of life of patients 19,38,41. There is also an association of olfactory rehabilitation with that of the palate, which implies a gain for the oral feeding of the patient 38.

Olfactory rehabilitation is performed for the restoration of nasal flow, which is interrupted by the disconnection of the upper inferior pathway, preventing retronasal stimulation of olfactory receptors 17.

Loss of nasal function leads to thickening and desquamation of the mucosa due to irritation, excessive secretions and lack of air, and the use of a heat exchanger and humidity helps to significantly decrease the frequency of coughing, forced sputum and lung symptoms 21-25. The use of a heat exchanger is considered the gold standard in many countries 25.

Nutritional support is also a concern for these patients, as several studies have observed that adequate nutrition is a quality-of-life factor 26,31. Patients who are dependent on a feeding tube report lower quality of life 42. In Australia, dysphagia was observed in 90% of cases during hospital discharge, with a great social impact on the patient's quality of life. Given this observation, surgical techniques have been developed to minimize sequelae, initiating rehabilitation even intraoperatively 31.

In general, the interdisciplinary team acts in a manner that optimizes treatment and rehabilitation, in addition to improving quality of life mainly in terms of the functionality of the patient with total laryngectomy, and addresses several aspects of the patient, depending on the individual demand of each 5,27.

## FINAL CONSIDERATIONS

This study enabled a characterization of the scientific research on interdisciplinary interventions in the perioperative rehabilitation of patients who underwent total laryngectomy. In total, 33 articles met the inclusion criteria and were part of this integrative review.

The evidence found pointed to more multidisciplinary interventions for postoperative rehabilitation.

A shortage of intervention studies that portrayed strong evidence was observed, since the majority of the studies found were descriptive, with level-of-evidence classifications of III, IV and VI, which are considered to be weak. We also observed a lack of scientific research both in Brazil and in Latin America, making a reflection on the subject necessary so that new research is developed and disseminated.

Although the interventions found in the literature are effective in the rehabilitation of the total laryngectomized patient, interdisciplinarity is not evidenced; however, interdisciplinarity is emphasized in these studies as a factor for improvement in terms of practical assistance and the quality of life of patients. Thus, health professionals should focus more on both the practical assistance of patients and the generation of research studies that address the perioperative rehabilitation of laryngectomized patients, namely, intervention studies that can be applied in clinical practice and that enable improvements to patient quality of life.

## AUTHOR CONTRIBUTIONS

Baia WRM and Nogueira PC designed the research. Fores JML, Rosa VM, Silva EPF, Guterres EO and Marcelino A performed the research and analyzed the data. Fores JML, Rosa VM and Kulcsar MAV wrote the manuscript.

## Figures and Tables

**Table 1:** Search strategy adopted.

DATA BASE	P	I	Co
		**Controlled descriptors**	
MeSH	Laryngectomy	Patient care team/education	Rehabilitation
		Patient care team/manpower	
		Patient care team/methods	
		Patient care team/utilization	
DeCS	Laryngectomy	Patient care team	Rehabilitation

**Source:** prepared by the researchers.

**Table 2:** Level of evidence and meaning.

Classification system for hierarchy of evidence for interventions/treatment questions	
Level I	Evidence for systematic review or meta-analysis of all randomized controlled trials
Level II	Evidence from well-designed randomized clinical trials
Level III	Evidence obtained from well-designed clinical trials without randomization
Level IV	Evidence from well-designed case-control and cohort studies
Level V	Evidence of systematic reviews of descriptive and qualitative studies
Level VI	Evidence from a single descriptive or qualitative study
Level VII	Evidence of the opinion of authorities and/or expert committee reports

**Source:** Adapted from Melmyk and Fineout-Overholt, 2005 (8).

**Figure 1:** Data collection flowchart.

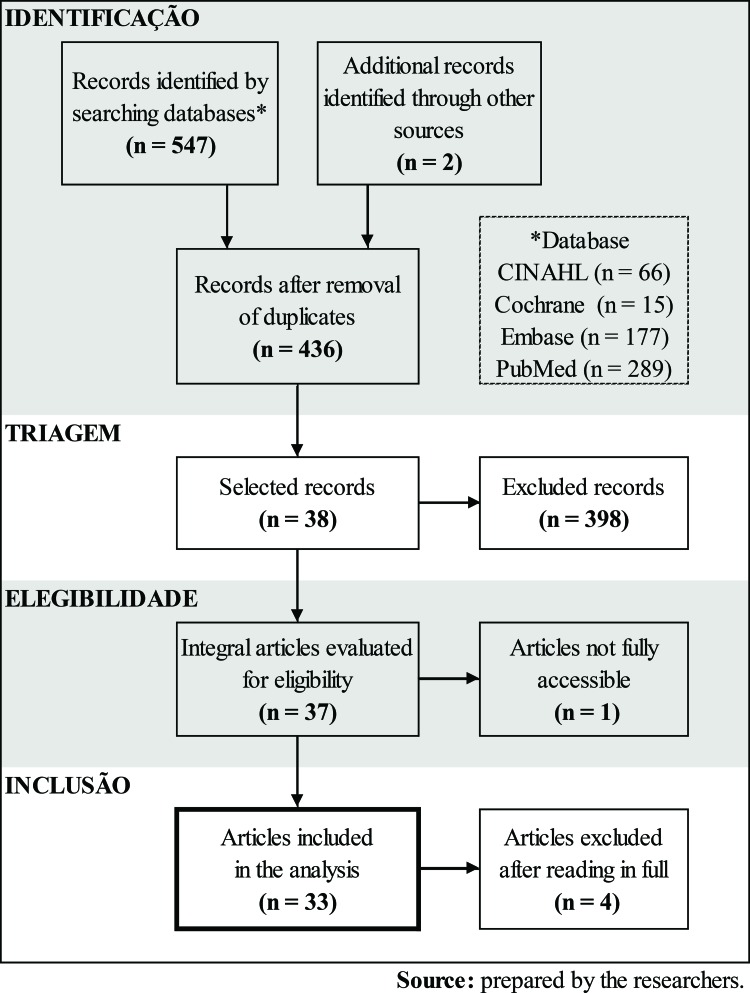

**Table 4:** Summary of articles and levels of evidence.

Title	Authors	Title of the journal	Intervention	Recommendations/Conclusion	Level of Evidence
The effect of a heat and moisture exchanger (Provox(¯) HME) on pulmonary protection after total laryngectomy: A randomized controlled study	Bien et al., 2010	European Archives of Oto-Rhino-Laryngology	Use of heat exchanger and humidity	There was a decrease in cough frequency, forced sputum, and stoma clearance after use.	II
Prospective clinical phase II study of two new indwelling voice prostheses (Provox Vega 225 and 20 Fr) and a novel anterograde insertion device (Provox Smart Inserter)	Hilgers et al., 2010	Laryngoscope	Voice for tracheoesophageal prosthesis	Voice characteristics were better in Vega 22.5, which coincides with patients' preference.	III
Olfactory rehabilitation after total laryngectomy	Morales-Puebla et al., 2010	Acta Otorrinolaringologica Espanola	Polite Yawning Technique	The polite yawning technique allowed an important recovery of smell and improvement of the palate after total laryngectomy.	III
Can laryngectomees smell? Considerations regarding olfactory rehabilitation following total laryngectomy	Moor et al., 2010	The Journal of Laryngology & Otology	Laryngeal derivation device; Polite Yawning Technique	The polite yawning technique had greater ease in teaching-learning.	VI
Rehabilitation of olfaction post-laryngectomy: A randomised control trial comparing clinician assisted *versus* a home practice approach	Ward et al., 2010	Clinical Otolaryngology	Polite yawning technique with professional-assisted training; Educated yawn technique with home training	The polite yawning technique, regardless of being accompanied by a professional or being trained at home, had effective olfactory rehabilitation, and the group assisted by the professional showed improvement in a shorter time.	II
Effectiveness of voice rehabilitation on vocalisation in postlaryngectomy patients: a systematic review	Xi, 2010	International Journal of Evidence-Based Healthcare	Voice by tracheoesophageal prosthesis; esophageal voice; voice by electrolarynx	The tracheoesophageal voice proved to be the best in terms of patient intelligibility and satisfaction.	V
Botulinum toxin type A: An effective treatment to restore phonation in laryngectomized patients unable to voice	Bartolomei et al., 2011	Neurological Sciences	Voice by tracheoesophageal prosthesis; esophageal voice; application of botulinum toxin type A	Treatment with botulinum toxin type A demonstrated efficacy in voice restoration for laryngectomy patients who were unable to perform vocal expressions because of spasms.	IV
Randomised, multi-centre study of the usefulness of the heat and moisture exchanger (Provox HME) in laryngectomised patients	Dassonville et al., 2011	European Archives of Oto-Rhino-Laryngology	Voice for tracheoesophageal prosthesis with heat and humidity exchanger	The prosthesis with heat and humidity exchanger improved the breathing pattern and vocalizations of laryngectomized patients.	II
Satisfaction and Quality of Life in Laryngectomees after Voice Prosthesis Rehabilitation	Giordano et al., 2011	Folia Phoniatrica et Logopaedica	Voice by tracheoesophageal prosthesis	The implantation of voice prostheses in patients showed positive effects on quality of life in all laryngectomized patients.	IV
A prospective randomized multicenter clinical trial of the Provox2 and Groningen Ultra Low Resistance voice prostheses in the rehabilitation of post-laryngectomy patients: A lifetime and preference study	Harms et al., 2011	Oral Oncology	Voice for tracheoesophageal prosthesis	There was no significant difference between the prostheses, but the authors recommended PROVOX2, according to patient preference.	II
The significance of rhinomanometry in evaluation of postlaryngectomy olfactory rehabilitation by polite yawning technique	Manestar et al., 2011	Rhinology	Polite yawning technique	Olfactory rehabilitation should be taken as an integral part of the total post-laryngectomy rehabilitation program.	IV
Voice-related quality of life (V-RQOL) outcomes in laryngectomees	Moukarbel et al., 2011	Head and Neck	Voice by tracheoesophageal prosthesis; esophageal voice; voice by electrolarynx	The gold standard for voice rehabilitation is the tracheoesophageal prosthesis.	VI
Clinical use of a neck brace to improve hands-free speech in laryngectomized patients	Dirven et al., 2012	The American laryngological	Voice by tracheoesophageal prosthesis fixed with silicone adhesive; Voice for tracheoesophageal prosthesis fixed with silicone adhesive and external cervical collar	Voice by tracheoesophageal prosthesis fixed with a silicone adhesive and external cervical collar presented better results with hands-free speech.	II
Long-term oral intake through a salivary bypass tube with chronic pharyngocutaneous fistula	Gooi e Richmon, 2012	American Journal of Otolaryngology-Head and Neck Medicine and Surgery	Salivary tube technique	The salivary tube technique facilitated healing of the wound and contributed to the maintenance of oral feeding in persistent fistulas.	VI
Amount of airflow required for olfactory perception in laryngectomees: A prospective interventional study	Manestar et al., 2012	Clinical Otolaryngology	Polite yawning technique	Smell was rehabilitated after restoration of the nasal airflow with the educated yawn technique.	IV
Randomized controlled trial on postoperative pulmonary humidification after total laryngectomy: External humidifier *versus* heat and moisture exchanger	Mérol et al., 2012	Laryngoscope	Use of heat exchanger and humidity	The heat and moisture exchanger could be considered the best option for airway humidification in the immediate postoperative period of total laryngectomy.	II
Practice of laryngectomy rehabilitation interventions: a perspective from Hong Kong	Chan, 2013	Current Opinion in Otolaryngology & Head & Neck Surgery	Voice by tracheoesophageal prosthesis; esophageal voice; voice by electrolarynx	The most popular speech restoration method was tracheoesophageal puncture; however. laryngeal speech often failed to achieve significant tonal variations for Cantonese patients.	VI
Practice of laryngectomy rehabilitation interventions: a perspective from India	Chaukar et al., 2013	Current Opinion in Otolaryngology & Head & Neck Surgery	Voice by tracheoesophageal prosthesis	The practice of vocal rehabilitation by tracheoesophageal prosthesis is well established. However, local constraints pose distinct challenges in India, leading to innovative interventions to improve device performance and lifespan.	VI
Ultrasound-guided botulinum toxin injection: A simple in-office technique to improve tracheoesophageal speech in postlaryngectomy patients	Chaukar et al., 2013	Head and Neck	Voice by tracheoesophageal prosthesis with botulinum toxin type A	The patient was able to speak better for the tracheoesophageal prosthesis after the injection of botulinum toxin.	VI
Post laryngectomy speech rehabilitation outcome in elderly patients	Cocuzza et al., 2013	European Archives of Oto-Rhino-Laryngology	Voice for tracheoesophageal prosthesis in young and old	The possibilities of tracheoesophageal recovery in elderly patients did not present differences in comparison with younger patients.	III
International practice of laryngectomy rehabilitation interventions: a perspective from South Africa	Fagan et al., 2013	Current Opinion in Otolaryngology & Head & Neck Surgery	Voice by tracheoesophageal prosthesis; stoma coverage with cloth	Excellent results of voice rehabilitation with tracheoesophageal prosthesis could be obtained in a scenario of qualified and dedicated services in speech therapy, even in developing countries.	VI
Pulmonary rehabilitation after total laryngectomy: A randomized cross-over clinical trial comparing two different heat and moisture exchangers (HMEs)	Herranz et al., 2013	European Archives of Oto-Rhino-Laryngology	Use of heat exchanger and humidity	The use of a heat exchanger and humidity provided better humidification conditions and caused less discomfort for the study population.	II
Laryngectomy rehabilitation: a perspective from the United States of America	Hinni e Crujido, 2013	Current Opinion in Otolaryngology & Head & Neck Surgery	Voice by tracheoesophageal prosthesis; esophageal voice; voice by electrolarynx	A multidisciplinary approach in the rehabilitation after laryngectomy discourse that covered the perioperative period produced better speech results.	VI
Practice of laryngectomy rehabilitation interventions: a perspective from Australia	Krishnan e Maclean, 2013	Current Opinion in Otolaryngology & Head & Neck Surgery	Voice by tracheoesophageal prosthesis; polite yawning technique	The method preferred by speech therapists for vocal rehabilitation and voice by tracheoesophageal prosthesis was olfactory rehabilitation	VI
Laryngectomy rehabilitation in the United Kingdom	Owen e Paleri, 2013	Current Opinion in Otolaryngology & Head & Neck Surgery	Voice by tracheoesophageal prosthesis; esophageal voice; voice by electrolarynx	All patients should be evaluated preoperatively by a speech therapist, clinical nurse and nutritionist. Access to the speech therapist should be continuous. Voice by tracheoesophageal prosthesis is preferred over other methods.	VI
Effectiveness of olfactory rehabilitation according to a structured protocol with potential of regaining pre-operative levels in laryngectomy patients using nasal airflow-inducing manoeuvre	Risberg-Berlin et al., 2013	European Archives of Oto-Rhino-Laryngology	Polite yawning technique	All patients returned to the preoperative olfactory level, and one patient reported improved olfaction compared to the preoperative level.	IV
Speech rehabilitation during the first year after total laryngectomy	Singer et al., 2013	Head and Neck	Voice by tracheoesophageal prosthesis; esophageal voice; intelligibility assessment	Failure to perform rehabilitation was associated with poorer functionality.	III
Practice of laryngectomy rehabilitation interventions: a perspective from Europe/the Netherlands	van der Molen et al., 2013	Current Opinion in Otolaryngology & Head & Neck Surgery	Voice by tracheoesophageal prosthesis; esophageal voice; voice by electrolarynx; use of heat exchanger and humidity; Polite yawning technique	Comprehensive multidisciplinary rehabilitation substantially decreased long-term morbidity and significantly benefited the quality of life of laryngectomized patients. The preferred method for reestablishing post-laryngectomy voice communication was the tracheoesophageal prosthesis.	VI
Practice of laryngectomy rehabilitation interventions: a perspective from South America	Vartanian et al., 2013	Current Opinion in Otolaryngology & Head & Neck Surgery	Voice by tracheoesophageal prosthesis; esophageal voice; voice by electrolarynx	The electrolaryngeal approach was considered the easiest method of vocal rehabilitation. The esophageal voice was considered the most difficult method but at a lower cost. Tracheoesophageal prosthesis was better in relation to quality of life and patient satisfaction.	VI
Changing trends of speech outcomes after total laryngectomy in the 21st century: A single-center study	Moon et al., 2014	The American Laryngological Association	Voice by tracheoesophageal prosthesis	There was no significant difference in voice rehabilitation with an independent tracheoesophageal prosthesis if the technique was started immediately after surgery versus later.	IV
Scientific evidence regarding the quality of life of total laryngectomees	Nemr col., 2015	Archives of Otolaryngology and Rhinology	Voice by tracheoesophageal prosthesis; esophageal voice; voice by electrolarynx	Patients rehabilitated with tracheoesophageal prosthesis presented better quality of life than those rehabilitated with other methods, but therapeutic success was achieved when the real needs and expectations of each individual were contemplated.	VI
Pulmonary rehabilitation after total laryngectomy	Parrilla et al., 2015	Annals of Otology, Rhinology & Laryngology	Use of heat exchanger and humidity	Quality of life improved with the reduction in pulmonary symptoms and significant influence of XtraHME on pulmonary status.	IV
Cost-effectiveness of heat and moisture exchangers compared to usual care for pulmonary rehabilitation after total laryngectomy in Poland	Retel et al., 2015	European Archives of Oto-Rhino-Laryngology	Use of heat exchanger and humidity	The use of a heat and humidity exchanger resulted in fewer lung infections and sleep problems, less use of external humidifiers in the hospital and a higher quality of life than usual care. The use of the exchanger in Poland was cost-effective.	III

**Source:** prepared by the researchers.
